# Assessment of Neurophysiological Parameters During Anterior Cervical Discectomy and Fusion and Their Correlation with Clinical Findings

**DOI:** 10.3390/jcm14082647

**Published:** 2025-04-12

**Authors:** Vedrana Karan Rakic, Djula Djilvesi, Djurdja Cvjetkovic Nikoletic, Tanja Lakic, Jelena Klasnja, Sonja Lukac Pualic, Mladen Karan

**Affiliations:** 1 Faculty of Medicine, University of Novi Sad, 21000 Novi Sad, Serbia; djula.djilvesi@mf.uns.ac.rs (D.D.); djurdja.cvjetkovic-nikoletic@mf.uns.ac.rs (D.C.N.); tanja.lakic@mf.uns.ac.rs (T.L.); jelena.klasnja@mf.uns.ac.rs (J.K.); 2 Center for Radiology, University Clinical Center of Vojvodina, 21000 Novi Sad, Serbia; sonja.lukac@mf.uns.ac.rs; 3 Department of Neurosurgery, Haukeland University Hospital, 5009 Bergen, Norway; mladen.karan@helse-bergen.no

**Keywords:** intraoperative neurophysiological monitoring, somatosensory-evoked potentials, motor-evoked potentials, anterior cervical discectomy and fusion, neck disability index, pain

## Abstract

**Background:** In this study, we used intraoperative neurophysiological monitoring (IONM) during anterior cervical discectomy and fusion (ACDF). Rather than emphasizing its use for safety purposes, our goal was to evaluate how neurophysiological parameters change during surgery and their correlation with clinical findings. **Methods:** This study included 30 patients who underwent ACDF. Detailed neurological examination was performed together with manual muscle testing (MMT), the Numeric Pain Rating Scale (NPRS), and the Neck Disability Index (NDI) questionnaire. During surgery, somatosensory-evoked potentials (SSEPs), motor-evoked potentials (MEPs), and spontaneous electromyography were registered. **Results:** There were statistically significant difference in the latency and amplitude of SSEPs of the right median nerve. Regarding the left median nerve, there was a statistically significant difference in amplitude, but not in latency. Differences were also observed in the amplitudes of right and left tibial nerve SSEPs, though no significant differences were found in their latencies. No statistically significant difference was found in the threshold values required to elicit MEPs between the beginning and end of the surgery. Additionally, we found a statistically significant positive correlation between the latency of the left and right median nerve and the left tibial nerve with somatosensory impairment. There was also a significant negative correlation between the amplitude of both tibial nerves and somatosensory impairment, and their latency showed a significant negative correlation with pain level before surgery. We found statistically significant decreases in NDI and pain level values one month after surgery. **Conclusions:** The results show significant changes in SSEPs and a correlation between clinical and neurophysiological findings and emphasize the importance of using MEPs to assess the condition of the motor system. Additionally, there was a general improvement in the patients’ condition, as assessed by NDI and pain scores. This study identifies critical surgical phases to consider in the absence of real-time neuromonitoring feedback and emphasizes that clinical observations may not fully reflect the condition of neurological structures in patients with myelopathy, which is crucial when deciding on timely surgery.

## 1. Introduction

Degenerative spinal diseases are a consequence of spondylotic changes in the spinal column, without the impact of trauma, infection, or other factors. These changes can be in the form of disc herniation, osteophyte formation, ligament hypertrophy, and ossification. Such alterations can cause the narrowing of the nerve root foramen and/or spinal canal. Degenerative changes in the spine can present clinically as radiculopathy (compression of the nerve root), myelopathy (compression of the spinal cord), or a combination of both. These conditions are the most common indications for surgical treatment with anterior cervical discectomy and fusion (ACDF).

The prevalence of degenerative cervical spine diseases has been rising, particularly in highly developed countries, where ageing has a great impact on their epidemiology [1]. This is not only a medical issue but has great socioeconomic impact. Between 2008 and 2014, cases of degenerative cervical spine diseases in the United States increased by 42% [2]. Similarly, Norway experienced a 74.1% rise in annual surgical rates for degenerative cervical spine diseases over the same period [3]. In China, a large retrospective study by Li et al. reported a prevalence of 49.6% for degenerative cervical spine diseases [4]. Additionally, surgical procedures for ACDF are projected to increase by 13.3% from 2020 to 2040 [5]. These data highlight the importance of providing adequate treatment for these patients.

Conservative treatment often provides adequate symptom relief in these patients. The indication for surgical treatment depends on the type and progression of neurological symptoms [6]. The most common surgical procedure used in the treatment of this group of patients is ACDF, which can provide adequate anatomical and functional restitution of degenerative cervical spine [7,8,9,10]. Intraoperative neurophysiological monitoring (IONM) provides real-time information about the integrity and function of neural structures [11,12,13]. Multimodal neuromonitoring, including the use of somatosensory-evoked potentials (SSEPs), motor-evoked potentials (MEPs), and electromyography (EMG), is widely used today and provides as comprehensive a picture as possible [14]. The most common application of SSEP is monitoring the spinal cord during surgeries for conditions such as scoliosis, cervical myelopathy, fractures, tumours, and other disorders that may put the spinal cord at risk. The indication for using MEP is any surgical procedure where there is a risk of damaging the motor pathways or primary motor cortex. This includes, but is not limited to, surgeries on the spine or spinal cord and orthopedic interventions such as procedures for deformities, bone tumours, spinal cord decompression, and trauma. Electromyography (EMG) records the electrical activity of muscles. It can be spontaneous, which registers spontaneous muscle activity and allows for continuous monitoring, or stimulated, which involves the direct stimulation of peripheral nerves or spinal roots. Surgical manipulation, such as pulling, stretching, or compression, activates specific muscles that can be detected through spontaneous electromyography.

The spinal cord and nerve roots may be at risk during ACDF, which may lead to new postoperative neurological deficits [15,16,17]. In recent years, there has been debate regarding the routine use of IONM during ACDF, primarily due to the low rate of complications and cost-effectiveness. An evaluation of the impact of IONM on the safety and costs of ACDF found that, while IONM was not associated with a reduced complication rate, it led to increased costs [18,19,20,21]. The utility of IONM in ACDF remains a subject of debate [19,22], yet its usage has been steadily increasing. The utilization of IONM has increased from 0.9% in 2007 to 36.7% in 2018 [22]. Even though these complications are rare [21,23,24], they are a devastating outcome of spinal surgery [25,26]. It is important that the information provided by IONM in patients undergoing surgery for degenerative cervical spine pathology is not undermined [13], although the routine use of IONM remains controversial [27].

Defining critical phases during surgery that may lead to neurological damage is essential, as is the ability to obtain real-time information which can influence intraoperative procedures, such as patient positioning. It is also important to recognize when changes meet alarm criteria, prompting timely interventions. The surgeon’s ability to make decisions based on real-time feedback is crucial. Additionally, gaining insight into the real condition of compromised neural structures is very important. The use of neuromonitoring facilitates the accomplishment of all these objectives.

While there is considerable research on the use of neurophysiological monitoring to detect changes which could potentially lead to neural damage and help prevent it, less is known about how specific surgical phases influence these neurophysiological signals. Most existing studies focus on the preoperative baseline or postoperative outcomes, with less attention given to how these parameters change throughout the different stages of surgery. In this context, our focus was not on evaluating the overall effectiveness of IONM in ACDF. Instead, this study aimed to examine how neurophysiological parameters change at different stages of the surgery, to determine whether neurophysiological findings correlate with clinical findings and provide better insight into the patient’s condition.

## 2. Materials and Methods

This one-year study included 30 patients from a single centre who underwent ACDF for degenerative changes in the cervical spine.

The inclusion criteria for this study were the following:

- Male and female patients, 18 years and older;
- Indications for surgical treatment of compressive cervical radiculopathy:
  - Progressive, significant neurological deficit;
  - Static neurological deficit accompanied by severe radicular pain;
  - Persistent or recurrent arm pain unresponsive to conservative treatment;
- Indications for surgical treatment of compressive cervical myelopathy:
  - Progressive spinal cord damage without permanent remission;
  - No improvement after conservative treatment for mild-form myelopathies;
- Signed informed consent.

The exclusion criteria for this study included the following:

- Presence of any motor deficit not caused by the patient’s primary conditions—cervical radiculopathy and/or cervical myelopathy;
- Previous hospitalization or outpatient treatment for diseases of the neuromuscular system (e.g., amyotrophic lateral sclerosis, diabetic polyneuropathy, alcoholic polyneuropathy, myasthenia gravis, muscular dystrophies, myopathies, etc.);
- Presence of sensory syndromes (e.g., dorsal root and ganglion lesions, complete transverse spinal cord injury, posterior funicular syndrome, Brown-Séquard syndrome, brainstem lesion syndrome, thalamic and sensory cortex lesion syndrome);
- Patients with chronic conditions that could impact IONM, such as diabetes, uncontrolled arterial hypertension, hypo- and hyperthyroidism, and lung diseases.

**Scheme 1:**
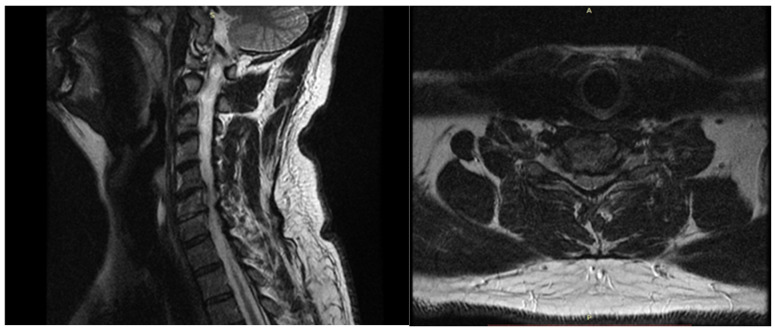
T2-weighted MRI images—sagittal and axial view of the cervical spine.

The preoperative evaluation included a detailed anamnesis and a review of complete medical records to ensure adherence to the study’s inclusion/exclusion criteria. All patients underwent an MRI (Scheme 1), but none had preoperative neurophysiological tests, such as EMG, SSEP, or MEP, as these are not part of the standard procedure. Preoperative assessments involved thorough neurological examination, manual muscle testing (MMT), the Numeric Pain Rating Scale (NPRS), and the Neck Disability Index (NDI) questionnaire. Postoperative assessments of pain and NDI were conducted one month after surgery. In all cases, we used multimodal IONM, which included somatosensory-evoked potentials (SSEPs), motor-evoked potentials (MEPs), and spontaneous electromyography (EMG).

For the stimulation and recording of SSEPs, MEPs, and EMG, the ISIS IOM portable system (Inomed Medizintechnik GmbH, Emmendingen, Germany) was used. SSEPs were elicited from median and posterior tibial nerves. The nerves were stimulated bilaterally in an alternating fashion using needle electrodes placed at defined points (frequency 4.7/3.7 Hz, pulse width 200 μs, intensity 20–40 mA). Registration was performed according to the International 10–20 system at the CPz, CP3, CP4, and Fz points using corkscrew electrodes. Transcranial electrical stimulation to elicit MEPs was performed using corkscrew electrodes placed at the Cz, C3, and C4 points (train of 5 pulses, ISI 4 ms, pulse width 500 μs, intensity 40–220 mA). Standard muscle responses were recorded bilaterally from the abductor pollicis brevis and tibialis anterior muscles, with additional recordings from clinically relevant muscles (e.g., deltoids, biceps, and triceps), depending on the level of surgery. Spontaneous EMG monitored muscle activity throughout the surgery. SSEPs and EMG were continuously monitored, while MEPs were assessed at key surgical stages in coordination with the surgeon, especially to address potential patient movement during transcranial stimulation. Six predefined points were analyzed: neutral position, positioning, approach to the spinal column, disc removal, implant placement, and post closure. We used total intravenous anesthesia (TIVA) exclusively, with a combination of propofol and remifentanil, without the addition of any inhalation anesthetics. The muscle relaxant rocuronium was used solely for induction and intubation. Since the temperature in the operating room is maintained between 20 and 21 degrees Celsius, standard procedure involves using a forced-air warming blanket to keep the body temperature within the physiological range. Body temperature, blood pressure, and arterial oxygen saturation were monitored throughout all procedures and maintained within the normal range.

Statistical analysis was conducted using the SPSS 20.0 software. Since most of the data did not follow a normal distribution, non-parametric tests were applied. To assess differences between repeated measurements of the variables, Friedman’s test was used, with subsequent analysis by Wilcoxon’s test (with Bonferroni’s correction for *p*-values). Correlations between the examined parameters were determined using Spearman’s rank correlation (Spearman’s ρ). A *p*-value of ≤ 0.05 was considered statistically significant.

## 3. Results

Regarding the gender distribution, 56.7% of patients were male, and 43.3% were female. The median age of the patients was 51 ± 10.5 years. The youngest patient was 30 years old and presented with symptoms of severe myelopathy, while the oldest was 64 years old and had symptoms of radiculopathy. In 60% of the patients, symptoms persisted for more than 12 months. The most commonly affected levels were C5-6 (62.2%), C6-7 (16.2%), C4-5 (16.2%), and C3-4 (5.4%). Neurological examination revealed a somatosensory impairment in 83.3% of patients. Reflex responses ranged from hyporeflexia to clonus, reflecting the heterogeneity of the sample. Manual muscle testing showed motor deficits in the upper extremities in 73.3% of patients, while motor deficits in the lower extremities were observed in 56.7%.

**Figure 1:**
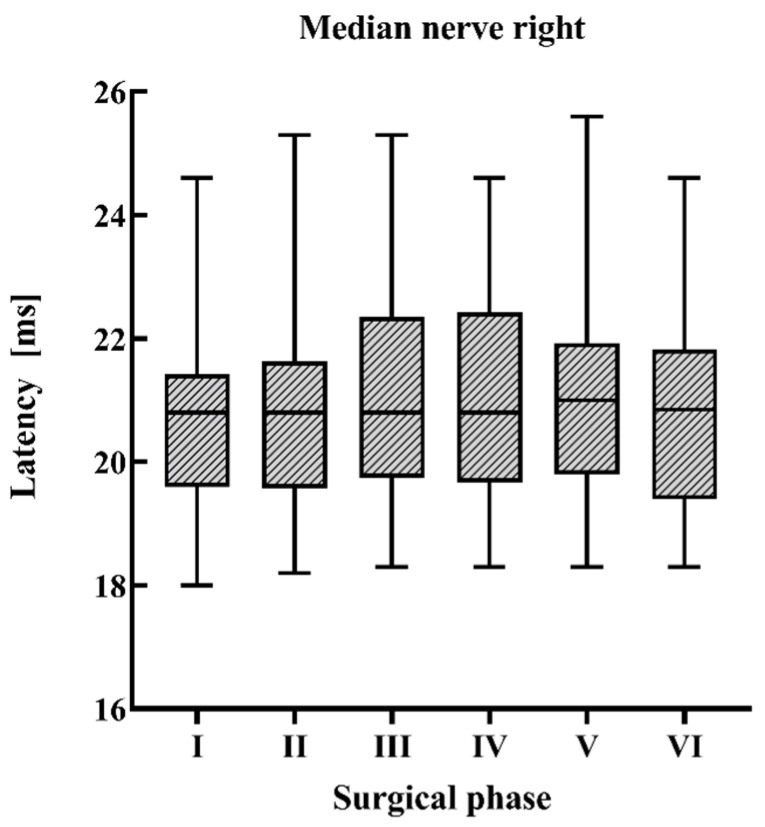
Latency of the right median nerve during surgery. I—neutral position, II—positioning, III—approach to the spinal column, IV—disc removal, V—implant placement, and VI—post closure.

**Figure 2:**
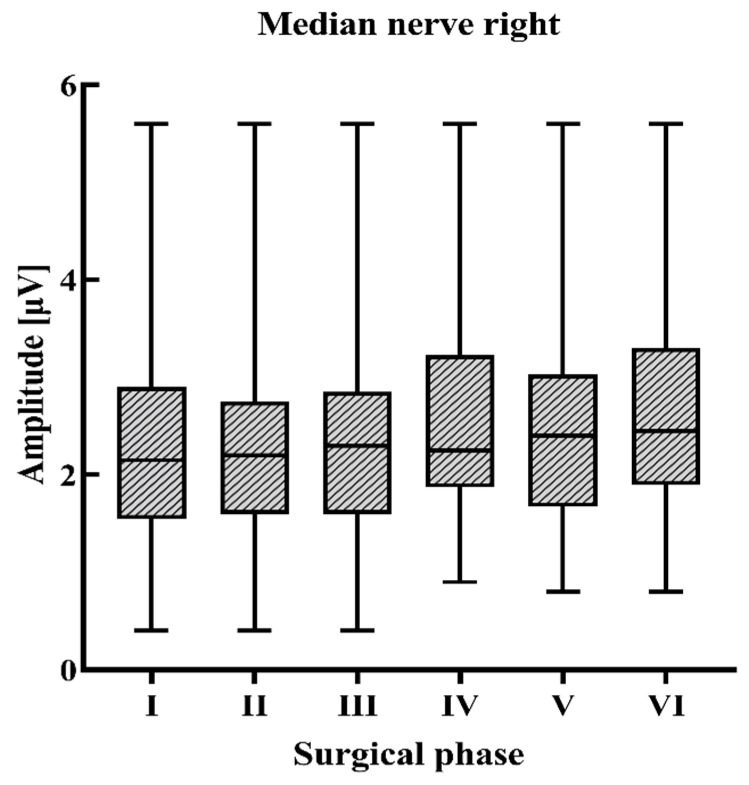
Amplitude of the right median nerve during surgery. I—neutral position, II—positioning, III—approach to the spinal column, IV—disc removal, V—implant placement, and VI—post closure.

There was a statistically significant difference (*p* < 0.05) in the latency and amplitude of the SSEP of the right median nerve (Figure 1 and Figure 2).

**Figure 3:**
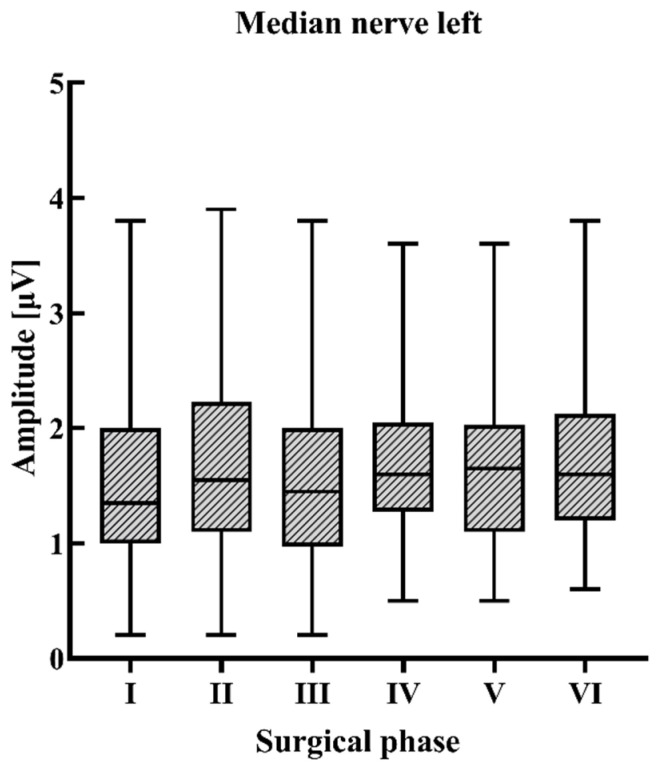
Amplitude of the left median nerve during surgery. I—neutral position, II—positioning, III—approach to the spinal column, IV—disc removal, V—implant placement, and VI—post closure.

**Figure 4:**
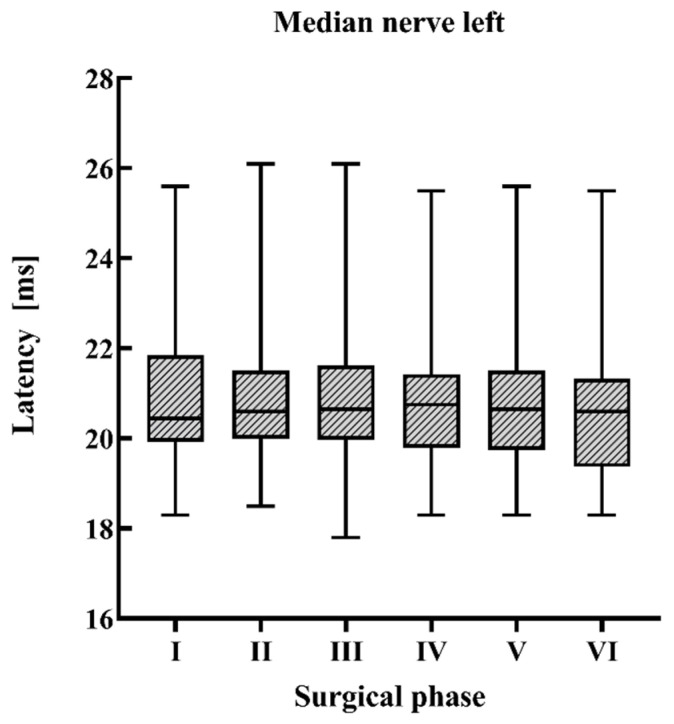
Latency of the left median nerve during surgery. I—neutral position, II—positioning, III—approach to the spinal column, IV—disc removal, V—implant placement, and VI—post closure.

Regarding the left median nerve, there was a statistically significant difference in amplitude (*p* < 0.05) (Figure 3) but not in latency (*p* > 0.05) (Figure 4).

**Figure 5:**
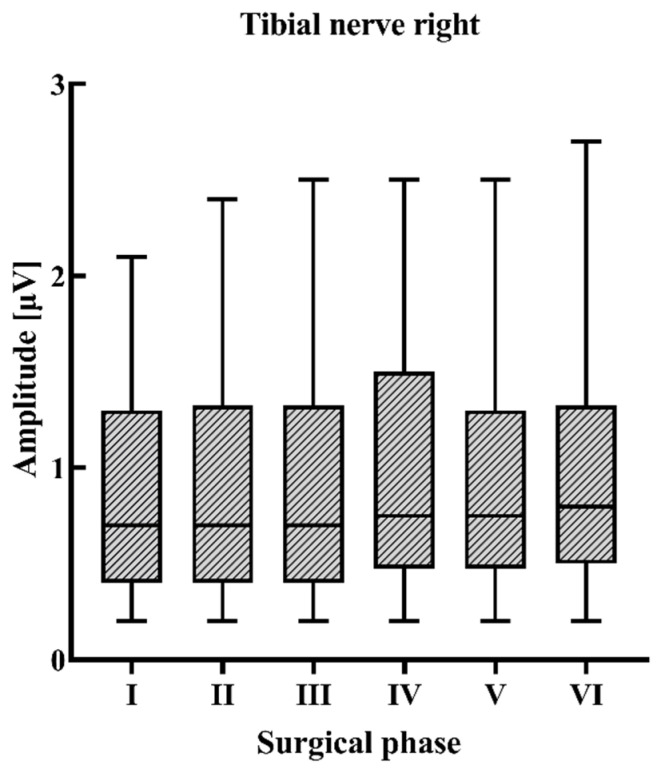
Amplitude of the right tibial nerve during surgery. I—neutral position, II—positioning, III—approach to the spinal column, IV—disc removal, V—implant placement, and VI—post closure.

**Figure 6:**
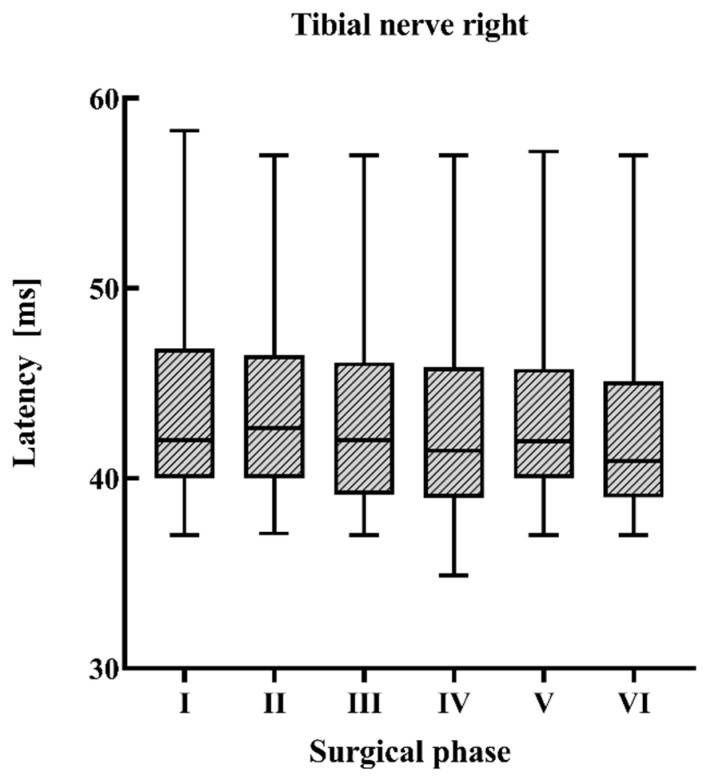
Latency of the right tibial nerve during surgery. I—neutral position, II—positioning, III—approach to the spinal column, IV—disc removal, V—implant placement, and VI—post closure.

**Figure 7:**
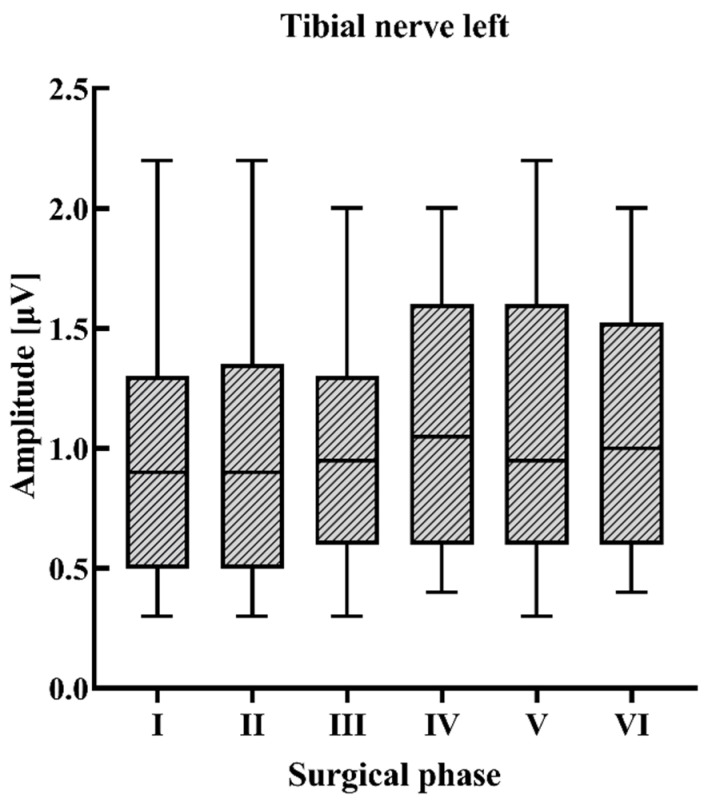
Amplitude of the left tibial nerve during surgery. I—neutral position, II—positioning, III—approach to the spinal column, IV—disc removal, V—implant placement, and VI—post closure.

**Figure 8:**
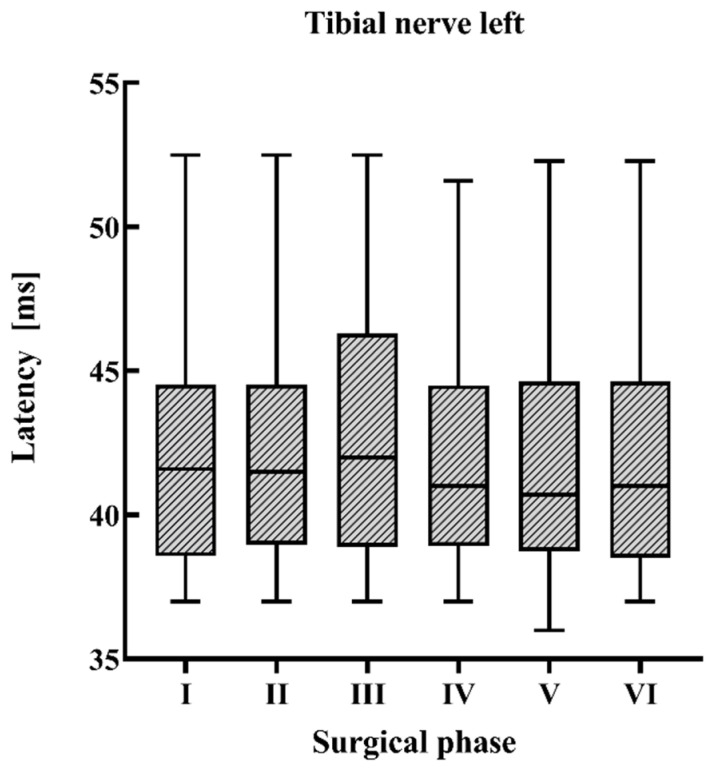
Latency of the left tibial nerve during surgery. I—neutral position, II—positioning, III—approach to the spinal column, IV—disc removal, V—implant placement, and VI—post closure.

A significant difference was also found in the amplitudes of SSEPs of the right and left tibial nerves (*p* < 0.05), although no difference was observed in the latencies (*p* > 0.05) (Figure 5, Figure 6, Figure 7 and Figure 8). In four patients with clinical signs of myelopathy, the SSEPs of the tibial nerve could not be recorded.

The results of the post hoc analysis, which was conducted to identify the phases of surgery during which the most frequent changes in neurophysiological parameters occur (with Bonferroni correction—statistical significance *p* ≤ 0.017), are presented in Table 1.

There was no statistically significant difference (*p* > 0.05) in the threshold values for eliciting MEPs between the beginning and end of surgery. In six patients with myelopathy, some standard MEPs could not be elicited either at the beginning or at the end of surgery. The continuous EMG did not detect any significant changes that would have indicated intraoperative impairment of the nerve roots.

Table 2 shows the correlation between somatosensory-evoked potentials (SSEPs), somatosensory impairment, and pain level before surgery. A statistically significant positive correlation (*p* < 0.05) was found between the latency of the left and right median nerves and the left tibial nerve with somatosensory impairment. Additionally, a significant negative correlation (*p* < 0.05) was observed between the amplitude of both tibial nerves and somatosensory impairment. The latency of both median and tibial nerves showed a significant negative correlation (*p* < 0.05) with the pain level before surgery.

No correlation was found between the MEPs and manual muscle testing (MMT).

**Figure 9:**
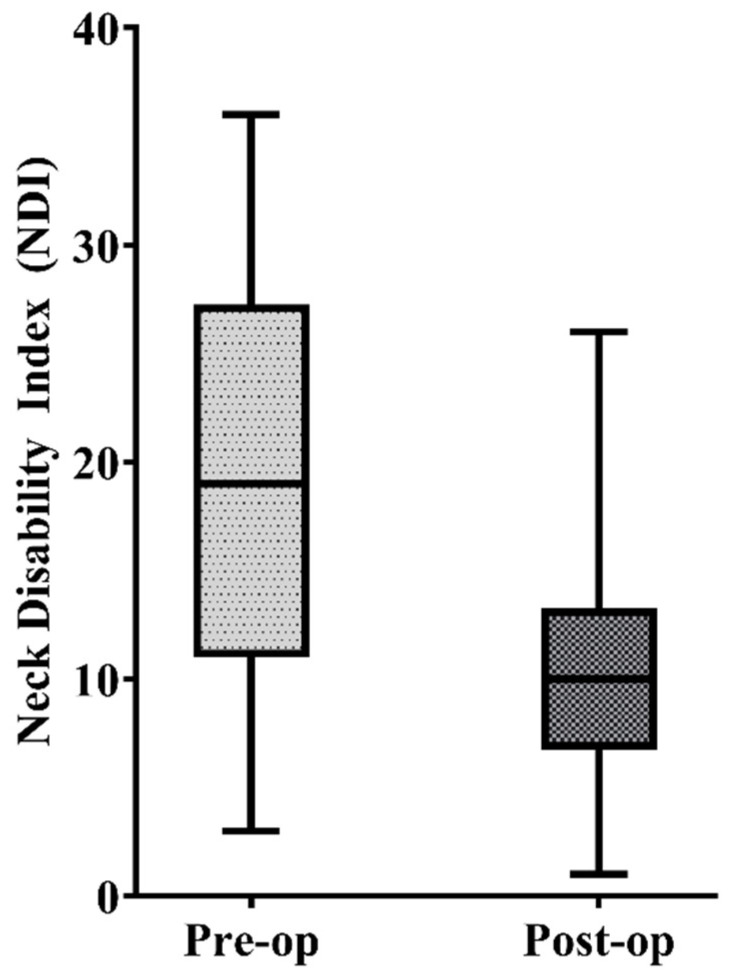
Neck Disability Index before surgery and one month after surgery.

Analysis of the Neck Disability Index (NDI) questionnaire revealed that most patients had mild (33.3%) or moderate (33.3%) disability, while 26.7% had severe disability. One patient (3.3%) had no disability, and one patient (3.3%) had complete disability. A statistically significant decrease in NDI scores was observed one month after surgery (*p* < 0.05) (Figure 9).

**Figure 10:**
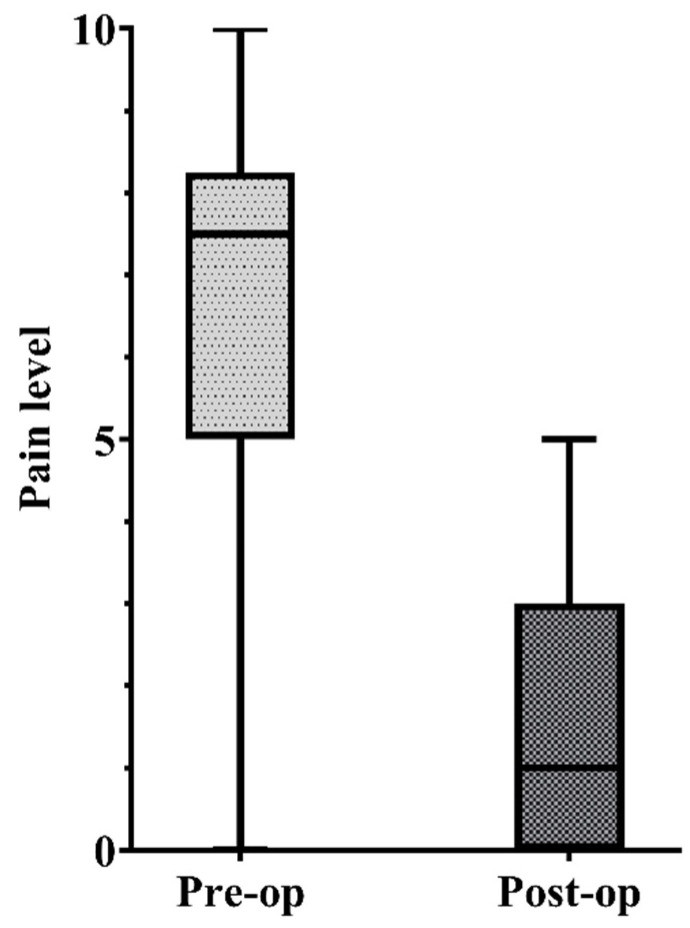
Pain level before and one month after surgery.

According to the Numeric Pain Rating Scale, 60% of patients reported severe pain, 26.7% reported moderate pain, 6.6% had mild pain, and two patients (6.6%) were without pain. Pain level also was significantly lower (*p* < 0.05) one month after surgery (Figure 10).

## 4. Discussion

Most research focuses on the utility of IONM in ACDF to prevent new neurological deficits and assess its cost-effectiveness. In our study, we did not emphasize its use for safety reasons, as these have already been extensively studied. Instead, we aimed to examine how neurophysiological parameters change during surgery—specifically, whether they are present at the onset of the surgery, how they change throughout the procedure, when these changes are most pronounced, whether they remain stable, and whether the effects of decompression are maintained. Additionally, we explored how clinical findings correlate with neurophysiological parameters, specifically whether clinical findings are reliable indicators of the severity of the condition. The use of neurophysiological tests is crucial as they assess function beyond the anatomy of the nervous system [28], yet preoperative testing is limited. In this context, IONM provides valuable insights into the function of compromised neural structures, as current clinical guidelines for spinal surgery recommend multimodal IONM as a reliable and valid method for assessing spinal cord integrity [29,30].

If there is damage to spinal cord function due to compression or ischemia, SSEPs typically change in proportion to the degree of damage. An increase in latency is a consequence of slowed conduction, which implicates a demyelination process. A decrease in amplitude indicates axonal loss. Compressive lesion leads to segmental demyelination, which can manifest as changes in both latency and amplitude. If the compressive effect that caused these changes in SSEPs can be removed and the damage of the spinal cord is not irreversible, both latency and/or amplitude can be improved after decompression. It was observed that surgical procedures leading to decompression and reduction in ischemia contribute to the improvement of these potentials [31]. Some authors claim that SSEPs may have a prognostic role because they estimate the integrity of the spinal cord [32,33], while others suggest that SSEPs can predict short-term improvement after surgery, but not long-term outcomes [34]. This study included patients with radiculopathy, myelopathy, or both, and findings in the neurophysiological parameters were consistent with their clinical condition. In our cohort, a significant increase in the amplitude of all SSEPs was registered, which may indicate a significant level of decompression. Wi et al. found that, in a group of 27 patients with compressive myelopathy, 13 demonstrated only intraoperative improvement in SSEPs and experienced significant improvements in neurological function and subjective symptoms after surgery [35]. In contrast, some authors have found that SSEPs respond slowly to spinal decompression and do not change significantly [36,37]. Although many patients who showed improvement in SSEP amplitude reported significant recovery on the NDI questionnaire and pain scale one month after surgery, we cannot conclusively state that SSEP improvement is a predictive factor. A longer follow-up period is necessary to determine whether SSEP improvements can serve as a predictor for long-term outcome. The potentially most critical stages in ACDF are the removal of the disc and the placement of the implant. The most frequent changes in neurophysiological parameters occurred during the disc removal phase due to the highest level of decompression. Most of these changes remained until the end of the surgery, which supports the fact that the effects of decompression are preserved after implant placement. The latency of SSEPs mostly did not change significantly, which might have been due to long-term compression and permanent damage to somatosensory tracts in patients with myelopathy. In four patients with myelopathy, SSEPs of the tibial nerve could not be registered. Two patients showed no response at either the beginning or end of the surgery, while in two other patients, SSEPs appeared after disc removal with longer latencies and lower amplitudes. Regarding motor-evoked potentials (MEPs), they remained stable during surgery. However, in six patients with myelopathy, some standard MEPs could not be elicited, which might have indicated the severity of the condition. Some authors consider MEPs of the lower extremities to be more sensitive in the detection of myelopathy, and there are differences in SSEPs and MEPs between patients with radiculopathy and those with myelopathy [38]. Although our study did not demonstrate a significant improvement in MEP, other authors have found that MEP could be a valuable predictive factor for recovery. Kombozo et al. found that an improvement in MEP during cervical myelopathy surgery is a highly predictive factor of early postoperative recovery and highlights the potential of MEP monitoring as a real-time predictive factor [39]. Wang et al. state that patients with intraoperative enhancement of MEP experience better early and long-term recovery [36]. Park et al. demonstrated that significant intraoperative MEP changes were indicative of functional improvement one month postoperatively; however, they did not serve as predictors of long-term outcomes [37].

The correlation between clinical assessment and neurophysiological parameters showed a significant correlation between somatosensory impairment and pain with SSEPs, which is consistent with the compressive effect on the spinal cord. However, no correlation was found between MEPs and MMT scores, which may imply that clinically preserved muscle strength is not a reliable indicator of the motor system condition. A possible reason for this finding is that MMT assesses overall muscle strength, while MEPs evaluate the integrity of the corticospinal pathway, meaning that subtle changes in neural function may not be detected by MMT. MEPs, on the other hand, may provide a more accurate reflection of the actual state. Simo at all. found that patients with mild clinical symptoms of myelopathy had abnormal MEPs and normal SSEPs, suggesting that MEPs may be more sensitive for detecting myelopathy in the early stage of disease [40]. These correlations may highlight the importance of neurophysiological testing to gain better insight into the functional status of the affected structures. Zdunczyk et al. report that cervical myelopathy results in functional reorganization at both the cortical and corticospinal levels. They propose a corticospinal reserve capacity model, suggesting that, in patients with mild myelopathy symptoms, adaptive mechanisms—such as the activation of supplementary motor areas and reduced cortical inhibition—enable patients to maintain functionality. However, the depletion of these mechanisms leads to the progression of more severe clinical symptoms [41]. This could perhaps explain the clinically preserved muscle strength observed in many patients, while MEPs reveal the true underlying condition.

Although the focus of this study was not on the efficacy of IONM in detecting and preventing new neurological deficits, IONM was certainly used for its primary purpose. In our study, there were no intraoperative changes in neurophysiological parameters that met alarm criteria, which would indicate potential damage. Additionally, no new neurological deficits were observed after surgery. Soda et al. state that IONM can help prevent neural injury and, when used alongside MRI, can serve as a predictor of neural recovery [42]. Wi et al. suggest that improvements in IONM signals are predictive of favourable neurological outcomes, particularly in spinal cord decompressive surgery [35].

The effectiveness of treatment in spinal surgery is often estimated using patient-reported outcome questionnaires, and some of the most commonly used questionnaires are the NDI and various pain scales [43]. Peolsson et al. suggest that NDI may be considered an important short-term and long-term outcome measurement in the evaluation of ACDF [44]. Although these questionnaires provide numerical values for disability and pain, their clinical meaning is not always clear [43]. To overcome this issue, the concept of the minimum clinically important difference (MCID) was introduced. The MCID represents the smallest change in outcome measure that is considered relevant for a patient [45]. There are several methods for calculating the MCID, resulting in a large variability in its reported values. The study by Parker et al. was the first to evaluate the MCID specifically related to ACDF and included only patients with radiculopathy [43]. They reported the following MCID values: 8 points for the NDI, 2.6 for neck pain, and 4.1 for arm pain. Carreon at al., in a study of 505 patients who underwent surgery for degenerative cervical spine conditions, calculated that patients whose NDI score decreased by 8 points and pain by 3 had MCID [46]. If we were to apply the MCID values from this study, considering the heterogeneity of our sample, we would find that, one month after surgery, 67% of patients had the MCID for NDI and 93% for pain.

The limitations of this study were undoubtedly the small sample size, which might have affected the generalizability and statistical power of the findings, and the heterogeneity of the sample, which may have introduced variability and affected the consistency of the results. A larger sample with patients clearly divided into two subgroups (patients with radiculopathy and patients with myelopathy) would greatly enhance the explanation of the results and help determine in which cases the use of IONM in ACDF is justified.

## 5. Conclusions

The results showed significant changes in SSEPs and a correlation between clinical and neurophysiological findings, as well as emphasized the importance of using MEPs to assess the condition of the motor system. Additionally, there was a general improvement in the patients’ condition, as assessed by the NDI and pain scores. In this study, IONM provided valuable insights into the functional status of the affected structures and the changes in neurophysiological parameters following decompression. The results of this study highlight critical phases of the surgical procedure that should be considered in the absence of neuromonitoring, which provides real-time feedback. Additionally, the findings suggest that clinical observations may not fully reflect the condition of the affected neurological structures in patients with myelopathy, a factor which must be considered when deciding on timely surgical intervention. This study could open the question of a more detailed preoperative neurophysiological assessment, which would more reliably reflect the true condition of compromised neural structures and, in turn, enable a more personalized approach in terms of the need for IONM in this procedure. Future research should focus on larger, multicenter studies with a homogeneous sample of patients diagnosed with myelopathy. These studies should aim to assess potential correlations between variables such as symptom duration, radiological findings (MRI, DTI), and the severity of myelopathy with IONM findings. Additionally, research should explore whether IONM can serve as a predictive tool for both short-term and long-term outcomes in these patients.

## Figures and Tables

**Table 1:** Results of post hoc analysis.

SSEP	Baseline/ Disc Removal	Baseline/ Implant Placement	Baseline/ Post Closure
Right median nerve latency	0.01 *	0.04	0.808
Right median nerve amplitude	0.001 *	0.02	0.002 *
Left median nerve latency	0.7	0.23	0.033
Left median nerve amplitude	0.016 *	0.29	0.006 *
Right tibial nerve latency	0.24	0.77	0.204
Right tibial nerve amplitude	0.015 *	0.07	0.008 *
Left tibial nerve latency	0.95	0.81	0.673
Left tibial nerve amplitude	0.09	0.15	0.003 *

* Statistical significance *p* ≤ 0.017.

**Table 2:** Correlation between somatosensory-evoked potentials (SSEPs), somatosensory impairment, and pain level prior to surgery.

SSEP	Somatosensory Impairment	Pain Level Before Surgery
Right median nerve latency	ρ 0.450 *	ρ −0.502 *
Right median nerve amplitude	ρ −0.119	ρ 0.189
Left median nerve latency	ρ 0.481 *	ρ −0.372 *
Left median nerve amplitude	ρ −0.072	ρ 0.292
Right tibial nerve latency	ρ 0.160	ρ −0.471 *
Right tibial nerve amplitude	ρ −0.436 *	ρ 0.196
Left tibial nerve latency	ρ 0.414 *	ρ −0.391 *
Left tibial nerve amplitude	ρ −0.380 *	ρ 0.179

* Statistically significant correlation (*p* < 0.05).

## Data Availability

The data presented in this study are available from the corresponding author upon reasonable request. The data are not publicly available due to privacy reasons.

## Abbreviations

The following abbreviations are used in this manuscript:

IONM	Intraoperative Neurophysiological Monitoring
ACDF	Anterior Cervical Discectomy and Fusion
MMT	Manual Muscle Testing
NPRS	Numeric Pain Rating Scale
NDI	Neck Disability Index
SSEPs	Somatosensory-Evoked Potentials
MEPs	Motor-Evoked Potentials
EMG	Electromyography
TIVA	Total Intravenous Anesthesia
MCID	Minimum Clinically Important Difference
